# Higher retention and viral suppression with adolescent-focused HIV clinic in South Africa

**DOI:** 10.1371/journal.pone.0190260

**Published:** 2017-12-29

**Authors:** Brian C. Zanoni, Thobekile Sibaya, Chelline Cairns, Sara Lammert, Jessica E. Haberer

**Affiliations:** 1 Massachusetts General Hospital, Boston, Massachusetts, United States of America; 2 Harvard Medical School, Boston, Massachusetts, United States of America; 3 University of KwaZulu-Natal Nelson Mandela School of Medicine, Durban, South Africa; 4 Don McKenzie Hospital, Botha’s Hill, South Africa; 5 University of Minnesota, Minneapolis, Minnesota, United States of America; University of New South Wales, AUSTRALIA

## Abstract

**Objective:**

To determine retention in care and virologic suppression among HIV-infected adolescents and young adults attending an adolescent-friendly clinic compared to those attending the standard pediatric clinic at the same site.

**Design:**

Retrospective cohort analysis.

**Setting:**

Government supported, hospital-based antiretroviral clinic in KwaZulu-Natal, South Africa.

**Participants:**

Two hundred forty-one perinatally HIV-infected adolescents and young adults aged 13 to 24 years attending an adolescent-friendly clinic or the standard pediatric clinic from April 2007 to November 2015.

**Intervention:**

Attendance in an adolescent-friendly clinic compared to a standard pediatric clinic.

**Outcomes measures:**

Retention in care defined as one clinic visit or pharmacy refill in the prior 6 months; HIV-1 viral suppression defined as < 400 copies/ml.

**Results:**

Overall, among 241 adolescents and young adults, retention was 89% (214/241) and viral suppression was 81% (196/241). Retention was higher among those attending adolescent clinic (95%) versus standard pediatric clinic (85%; OR 3.7; 95% confidence interval (CI) 1.2–11.1; p = 0.018). Multivariable logistic regression adjusted for age at ART initiation, gender, pre-ART CD4 count, months on ART, and tuberculosis history indicated higher odds of retention in adolescents and young adults attending adolescent compared to standard clinic (AOR = 8.5; 95% CI 2.3–32.4; p = 0.002). Viral suppression was higher among adolescents and young adults attending adolescent (91%) versus standard pediatric clinic (80%; OR 2.5; 95% CI 1.1–5.8; p = 0.028). A similar multivariable logistic regression model indicated higher odds of viral suppression in adolescents and young adults attending adolescent versus standard pediatric clinic (AOR = 3.8; 95% CI 1.5–9.7; p = 0.005).

**Conclusion:**

Adolescents and young adults attending an adolescent-friendly clinic had higher retention in care and viral suppression compared to adolescents attending the standard pediatric clinic. Further studies are needed to prospectively assess the impact of adolescent-friendly services on these outcomes.

## Introduction

In 2013, an estimated 870,000 adolescents and young adults aged 15–24 years were living with HIV in South Africa.[1] Depression/anxiety, perceived stigma, behavioral and conduct problems are common during adolescence and pose potential barriers to HIV care.[2–6] As adolescents age, they face developmental challenges, decreasing parental/guardian support, and experimentation with substance use that may act as additional barriers to care.[5, 7, 8] Changing care providers, rigid scheduling, increasing responsibilities, and decreasing involvement of adult caregivers may further contribute to the challenges of HIV care for adolescents.[7, 9]

To sustain viral suppression and achieve clinical benefits while receiving antiretroviral therapy (ART), individuals living with HIV/AIDS must remain engaged in medical care and maintain high adherence to medical regimens.[10] Numerous observational cohort studies in South Africa report significantly poorer retention in care and viral suppression rates for adolescents compared to older adults.[11–20] The World Health Organization (WHO) recently issued guidelines on provision of adolescent-friendly services to help overcome many of the barriers noted above to improve these outcomes;[21] however, there is little evidence of documented benefit from these services.[22, 23] A qualitative study with healthcare providers suggested that peer support and collaboration with healthcare providers may improve care for older HIV-infected adolescents and young adults in sub-Saharan Africa;[24] however, studies with objective outcomes are needed.

In this paper, we present an analysis of retention and viral suppression among adolescents attending an adolescent-friendly clinic compared to adolescents and young adults attending the same site’s standard pediatric clinic.

## Methods

### Setting

Don McKenzie Hospital is a regional tuberculosis hospital located in the Valley of 1,000 Hills, 35 kilometers west of Durban, KwaZulu-Natal, South Africa. Ethembeni Clinic is the associated HIV clinic located on the grounds of Don McKenzie Hospital. The HIV clinic opened in April 2007 and cares for >2,500 HIV-infected adults, >400 HIV-infected children (age birth to 12 years) and 241 HIV-infected adolescents and young adults (age 13–24 years). In the standard pediatric clinic, HIV-infected children are seen by the doctor and counselors on weekdays every 1–3 months and obtain medication the same day at an onsite pharmacy.

### Adolescent clinic

In March 2009, a Saturday adolescent clinic opened at the Ethembeni Clinic that included ART dispensing, lunch, and scheduled group activities (e.g., dancing, soccer, education, counseling). The clinic was established with the intention of decreasing school absenteeism and stigma and improving peer support and retention in care. Appointments at the Adolescent Clinic are every two months. Parents or caregivers are not required to attend. Initially adolescents over age 13 could attend if they were HIV-infected, fully aware of their HIV status, and receiving ART for 6 months. The six-month time frame allowed for prepackaging of weight-based ART medication during the time of anticipated rapid weight gain after ART initiation. Due to limited space and funding, the adolescent clinic was closed to new enrollment after reaching 80 adolescents in November 2012 and subsequent adolescents remained in the standard pediatric clinic until additional space in the adolescent clinic became available (e.g., when an older adolescent transitioned to adult care). Adolescents not attending the adolescent clinic remained in the standard weekday pediatric clinic. The same physician, nurses, and counselors work in both the adolescent clinic and pediatric clinic at the same clinical site. Clinical personnel working in the adolescent clinic are permitted to subtract hours they worked during the weekend clinic from their regular workweek hours. Additional expenses for each clinic, including the food and activities, were provided by a local non-profit organization and cost $1.25 per adolescent.

### Study design

We performed a retrospective cohort analysis of perinatally HIV-infected adolescents and young adults ages 13–24 years receiving at least one prescription of ART at Ethembeni Clinic from April 2007 to November 2015. Adolescents and young adults who initiated ART while hospitalized for tuberculosis and subsequently transferred to alternate facilities at discharge were excluded from the analysis. Any adolescent or young adult who attended the adolescent clinic at least once was considered exposed to the adolescent clinic for the primary analysis.

### Study procedures

We obtained demographic data, pre-ART information, medication history, pharmacy refill data, clinic visits, tuberculosis history, CD4, and viral load from medical records. We then compared retention in care and viral suppression among adolescents and young adults attending the two clinics. Retention in care was defined as one clinic visit and/or ART dispensing in the six months prior to data extraction (November 2015). All adolescents and young adults who were not retained were tracked by phone call, home visit, or a National Department of Health laboratory database search to ascertain their current status and to evaluate for undocumented transfers to alternate clinics. Viral suppression was defined as viral load <400 copies/ml from the most recent results (i.e., within the prior 6 months); missing viral load data were considered as not suppressed. Adolescents and young adults who died were considered not retained in care and not virally suppressed. Patients with documented transfers out of Ethembeni Clinic were considered retained in care if they had a clinic visit or ART dispensed elsewhere, or a viral load within three months of documented transfer. We performed a secondary analysis among all adolescents using the composite outcome of retained and suppressed defined as one clinic visit, pharmacy refill, and viral load <400 copies/ml within the previous 12 months in accordance with the South African National Treatment Guidelines.[25] Data were entered into a REDCap database. We used SAS version 9.4 (Cary, NC) to calculate descriptive statistics and conduct univariable and multivariable logistic regression models to assess retention in care and viral suppression. We included age at ART initiation, gender, ART duration, pre-ART CD4 (most recent CD4 prior to ART initiation), history of tuberculosis and adolescent clinic versus standard clinic in our models because these factors were previously shown to affect mortality, retention in care and viral suppression in similar South African pediatric HIV cohorts.[26–28] We performed secondary analyses including: evaluating transfers as not retained at the clinic; retention and viral suppression based on current clinic attendance to account for transfers back to the standard clinic from the adolescent clinic; retention in care over the last 3 months; and viral suppression among all active patients retained in care. In addition, we performed a sensitivity analysis excluding all adolescents and young adults who transferred care or were lost to follow-up within the first 6 months of treatment since they would not have been eligible to attend the adolescent clinic. To address potential confounding, we calculated a propensity score for adolescent clinic exposure controlling for age at ART initiation, gender, time on ART, pre-ART CD4, and history of tuberculosis. We included the propensity score in additional multivariable models to account for the chance of confounding by indication.[29] Propensity score matching reduces the chance of bias for those that may receive the intervention (adolescent clinic) due to covariates that may predict entry into the intervention group.

### Ethics statement

The Durban University of Technology Independent Review Committee, KwaZulu-Natal Department of Health and the Partners/Massachusetts General Hospital Research Ethics Board approved this protocol and granted a waiver of informed consent.

## Results

### Participants included in the analysis

**Fig 1:**
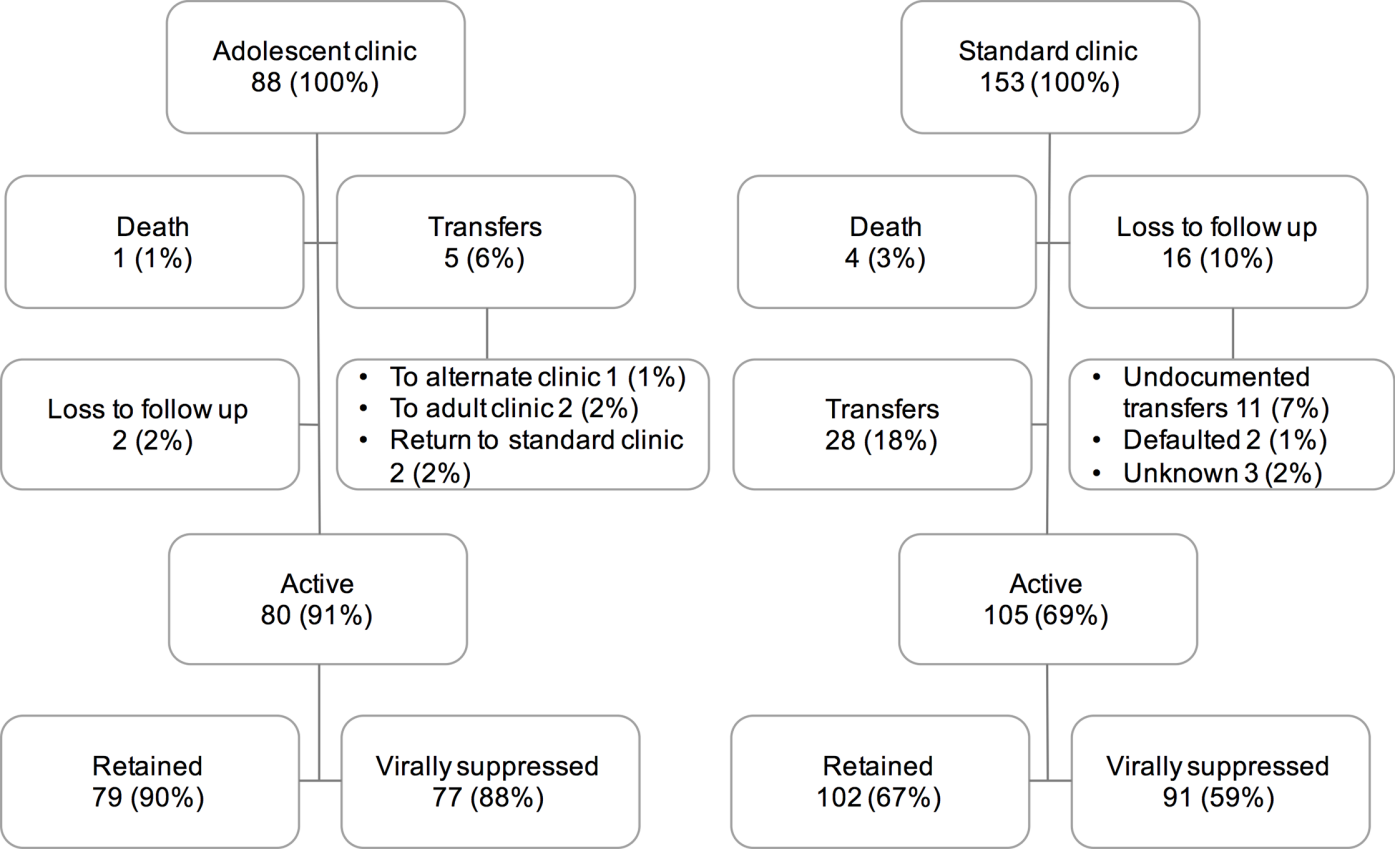
Flow diagram of retention in care and viral suppression in the adolescent clinic vs. the standard clinic.

A total of 254 adolescents and young adults ages 13–24 years received at least one prescription of ART from Ethembeni clinic between 2007 and 2015. Thirteen adolescents were excluded from this analysis because they initiated ART while hospitalized for tuberculosis at Don McKenzie Hospital and transferred to alternate facilities at discharge without attending the Ethembeni Clinic. The remaining 241 adolescents and young adults had a median of 67 months of follow-up (interquartile range [IQR] 40–84), of whom 88 attended the adolescent clinic at least once and 153 adolescents remained in the standard pediatric clinic. Among the entire cohort, a total of 29 (21%) adolescents transferred, 5 (4%) died, and 18 (13%) were lost to follow-up. Through participant tracking, 11 of those lost to follow-up were found in care at an alternate facility, 4 stopped ART (2 re-engaged in care and 2 declined to resume treatment), and 3 remained lost to follow-up; there were no known additional deaths. Comparing the adolescent clinic to the standard clinic 2% vs. 10% were lost to follow up; 1% vs. 3% died; 6% vs. 18% transferred, respectively as indicated in **Fig 1**. Of those attending the adolescent clinic, the median time in the adolescent clinic was 30 months (15 visits) with an IQR of 22 to 43 months (11 to 22 visits).

### Demographics

As shown in **Table 1**, the adolescents and young adults attending the adolescent clinic were significantly older at ART initiation (median 11.2 years; IQR 9.4–12.8 years) compared to those in the standard clinic (median 9.4 years; IQR 7.5–12.0 years; p = 0.002) and had significantly lower CD4 count at ART initiation (median 94 cells/mm^3^ vs. 211 cells/mm^3^; p<0.001). More adolescents and young adults attending adolescent clinic initiated a non-nucleoside reverse transcriptase inhibitor (NNRTI) versus protease inhibitor (PI) compared to those in the pediatric clinic (100% vs. 95%; p = 0.029). There were no differences in ART duration, current ART regimen, or history of tuberculosis.

**Table 1:** Characteristics of adolescents and young adults aged 13 to 24 years receiving antiretroviral therapy and attending an adolescent-friendly clinic or standard pediatric clinic from April 2007 to November 2015 in KwaZulu-Natal, South Africa.

	Adolescent clinic (n = 88)	Standard clinic (n = 153)	P-value
**Median age (years) at ART initiation (IQR)**	11.2 (9.4–12.8)	9.4 (7.5–12.0)	**0.002**
**Male (n)**	43% (38)	53% (80)	0.181
**Median age (years) at analysis (years; IQR)**	17.3 (15.5–18.4)	16.0 (13.7–17.5)	0.010
**Age (years) at entry to adolescent clinic (years; IQR)**	14.3 (13.2–15.4)	n/a	n/a
**Median pre-ART CD4 cells/mm^3^ (IQR)**	94 (35–220)	211 (110–353)	**<0.001**
**Initial ART: NNRTI (n)**	100% (88)	95% (145)	**0.029**
**Current ART: NNRTI (n)**	84% (74)	77% (118)	0.245
**Current ART: PI (n)**	16% (14)	23% (35)	0.245
**Median months on ART (IQR)**	80 (57–98)	74 (58–92)	0.246
**History of tuberculosis (n)**	48% (42)	54% (83)	0.351
**Retained in care (n)**	95% (84)	85% (130)	**0.018**
**Virally suppressed (n)**	91% (80)	80% (116)	**0.028**

Bold = p<0.05

Abbreviations: IQR–interquartile range; ART–antiretroviral therapy; NNRTI–Non-nucleoside Reverse Transcriptase Inhibitor; PI–Protease Inhibitor

### Retention

Overall, the retention rate was 89% (214/241). Through tracking we were able to determine current status outcomes for all adolescents and young adults except the 3 who remained lost to follow-up. We found significantly higher retention rates in adolescents and young adults attending the dedicated adolescent clinic (95%) versus those in standard care (85%; OR 3.7; 95% CI 1.2–11.1; p = 0.018). Multivariable logistic regression adjusting for age at ART initiation, gender, pre-ART CD4, months on ART, and history of tuberculosis indicated higher retention rates in adolescents and young adults attending the adolescent clinic compared to adolescents and young adults in the standard clinic (AOR = 8.5; 95% CI 2.3–32.4; p = 0.002). Younger age (AOR = 0.8; 95% CI 0.7–0.9; p = 0.010), males (AOR = 4.9; 95% CI 1.4–16.3; p = 0.010) and fewer years on ART (AOR = 0.8; 95% CI 0.6–1.0; p = 0.038) were also significantly associated with retention in care in this model **(Table 2).**

**Table 2:** Unadjusted and adjusted analysis comparing retention in care and viral suppression among adolescents and young adults attending an adolescent-friendly clinic compared to those attending standard pediatric clinic in KwaZulu-Natal, South Africa.

	Retention		Viral suppression	
	Unadjusted OR (P-value)	Adjusted OR (95% CI; P-value)	Unadjusted OR (P-value)	Adjusted OR (95% CI; P-value)
**Adolescent vs. standard clinic**	**3.7 (0.019)**	**8.5 (2.3–32.4; 0.002)**	**2.5 (0.031)**	**3.8 (1.5–9.7; 0.005)**
**Increasing age at ART initiation**	**0.8 (0.002)**	**0.8 (0.7–1.0; 0.010)**	**0.9 (0.051)**	0.9 (0.8–1.0; 0.086)
**Male**	**3.9 (0.005)**	**4.9 (1.5–16.3; 0.010)**	0.8 (0.515)	0.8 (0.3–1.8; 0.556)
**Increasing years on ART**	1.1 (0.353)	**0.8 (0.6–1.0; 0.038)**	1.0 (0.980)	0.9 (0.7–1.1; 0.271)
**Pre-ART CD4**	1.0 (0.393)	1.0 (0.9–1.0; 0.383)	1.0 (0.215)	1.0 (0.9–1.0; 0.132)
**Tuberculosis history**	0.6 (0.224)	0.8 (0.3–2.3; 0.741)	0.9 (0.692)	1.0 (0.4–2.2; 0.955)

Bold = p<0.05; Abbreviations: ART–antiretroviral therapy

### Viral suppression

Viral suppression results were available for 97% (233/241) of adolescents and young adults. Overall, the viral suppression rate was 81% (196/241). We found higher viral suppression rates among adolescents and young adults attending the adolescent clinic (91%) versus adolescents attending the standard pediatric clinic (80%; OR 2.5; 95% CI 1.1–5.8; p = 0.028). Multivariable logistic regression model adjusting for age at ART initiation, gender, pre-ART CD4, months on ART, and history of tuberculosis indicated higher viral suppression rates in adolescents and young adults attending adolescent clinic compared to those attending the standard pediatric clinic (AOR = 3.8; 95% CI 1.5–9.7; p = 0.005) (**Table 2**). No other factors were significantly associated with viral suppression in that multivariable model.

### Retained and suppressed within the last 12 months

We found that 73% (177) adolescents and young adults who initiated ART had the composite outcome of being in care with a suppressed viral load within the last 12 months. Multivariable logistic regression model adjusting for age at ART initiation, gender, pre-ART CD4, months on ART, and history of tuberculosis indicated higher retention with viral suppression in adolescents and young adults attending adolescent clinic compared to adolescents and young adults attending the standard pediatric clinic (AOR = 8.4; 95% CI 3.4–20.6; p<0.001).

### Secondary analyses

When considering all transfers as not retained at the Ethembeni clinic, adolescents and young adults attending the adolescent clinic were more likely to be retained in care (AOR 13.0; 95% CI 3.6–47.6; p = 0.0001) compared to adolescents and young adults attending the standard clinic. When considering current clinic attendance (accounting for transfers from the adolescent clinic back to standard clinic), adolescents and young adults currently attending the adolescent clinic were also more likely to be retained (OR 22.8; 95% CI 3.1–169.3; p = 0.002), suppressed (OR 5.4; 95%CI 1.8–15.8; p = 0.002) and retained and suppressed (OR 8.3; 95% CI 3.2–21.6; p < 0.0001) compared to those currently attending the standard clinic. Evaluating retention in care over the past 3 months, adolescents and young adults attending the adolescent clinic were more likely to be retained (OR 34.6; 95% CI 9.6–124.2; p < 0.0001) compared to adolescents and young adults attending the standard clinic. Viral suppression among active patients (excluding deaths, transfers, LTFU, and those not retained) was higher in the adolescents and young adults attending the adolescent clinic (OR 3.5; 95% CI 1.2–10.6; p = 0.03) compared to the standard clinic. Year of ART initiation was not significantly associated with retention or viral suppression and did not alter the primary outcome results.

### Sensitivity and propensity analyses

Compared to the primary findings, we found no significant differences in the sensitivity analysis excluding the 3 adolescents from the standard clinic who were not retained in care for the first 6 months of ART. Additionally, there was no difference in retention or viral suppression outcomes after including the propensity score in the final models.

## Discussion

Altogether, adolescents and young adults in this study had high rates of retention (89%) and viral suppression (81%). These findings are similar to a recent meta-analysis indicating an overall retention rate among HIV-infected South African adolescents of 83% and overall viral suppression rate of 81%.[30] In our cohort, despite having lower pre-ART CD4 and older age at ART initiation, adolescents and young adults attending the dedicated adolescent clinic had even higher retention (97%) and viral suppression rates (91%) than have been described in other South African adolescent HIV cohorts.[30]

Definitions of adolescent-friendly services vary and effective mechanisms likely hinge on convenient scheduling and peer support. The WHO recommends that adolescent-friendly services be accessible, acceptable, equitable, appropriate, and effective.[21] Tanner *et al* propose that the most important factors in adolescent-friendly services within the United States Adolescent Medicine Trials Network for HIV/AIDS Interventions (ATN) are the physical space and the social environment.[31] In our study, the physical environment and clinical staff were the same in each group. One major difference was in the accessibility by opening clinic on Saturdays. Adolescent-friendly services including afternoon and weekend clinic appointment can mitigate school absenteeism, decrease stigma of leaving school on a regular basis, and decrease wait times.[32–34] Combining medication collection with a social support/adherence group also decreases transportation costs and school absences by limiting trips to the clinic for both services. For the older adolescents, adolescent clinics present an opportunity to separate from parents and attend clinic alone in preparation for transition to the adult clinic. However, other settings have seen higher risk of viral failure in adolescents without parental involvement.[35] Adolescent-friendly clinics also provide a supportive social environment where peers can interact for emotional support.[31] Structured peer group therapy has been shown to decrease negative perception of illness, worries about illness, and improve viral outcomes particularly after sustained peer involvement.[36] The combination of peer support, youth focused clinic providers, and convenient scheduling likely contribute to the benefits of adolescent-friendly clinics.[34]

Adherence clubs, similar to the model used in the adolescent clinic in our study, have been a popular method to improve adherence and retention in care. Stable virally suppressed adults attending adherence clubs have shown higher retention in care and higher viral suppression rates than those attending standard clinics.[37, 38] Although there are multiple models of adolescent adherence clubs that bring together HIV-infected youth, published results on retention and viral outcomes have been limited. [36]

In our cohort, older adolescents and young adults had lower retention in care adjusting for clinic attendance. This finding indicates that older youth were less likely to be retained in either the standard or adolescent clinics. This highlights the difficulty with preparations for transition to adult care for older adolescents and young adults who often decrease engagement in care.[39] In our multivariate analysis, males had higher retention in care compared to females but there was no difference in viral suppression between the sexes. Sex differences in have been seen in other cohorts and are likely multifactorial and related to local socioeconomic factors.[40–43] In addition, our cohort saw lower initial CD4 in those adolescents and young adults attending the adolescent clinic compared to the standard clinic. This finding is likely due to the temporal changes in the South African National Treatment Guidelines. Older adolescents and young adults were started on ART with lower CD4s due to older recommendation to start ART for CD4≤ 200 cells/μl. These adolescents were the ones to fill the adolescent clinic. When the guidelines changed younger adolescents were initiated onto ART at higher CD4 counts but the adolescent clinic had already reached capacity.

Data on effectiveness of youth-friendly services compared to standard care on patient outcomes are limited. Teasdale *et al* saw no difference in retention at 6 and 12 months for newly diagnosed youth utilizing youth-friendly services compared with youth prior to the implementation of youth-friendly services in Kenya.[44] Their study used a pre/post intervention design and only evaluated retention in care at 6 and 12 months after ART initiation. Our study used a retrospective design where adolescent were receiving ART for a median of 73 months. The Teasdale study may not have seen differences due to short follow-up time or temporal differences in the pre/post design. High initial retention and viral suppression rates in adolescents that decrease over time have been previously documented in southern Africa which could explain why no difference was seen at 6 and 12 months after ART initiation.[45]

This study has several limitations. First, it was a retrospective analysis of an adolescent clinical program and not initially intended for a research design. Because adolescents were not randomized into the clinics, it is possible that unmeasured confounders may have lead to higher retention and viral suppression among adolescents attending the adolescent clinic; however, our propensity analysis did not find any significant difference in overall outcomes when adjusting for propensity score. Another limitation is that adolescents were required to be on treatment for 6 months prior to entry into the adolescent clinic. Our sensitivity analysis excluding the three adolescents not retained in the first 6 months did not find a difference in retention or viral suppression rates, although the small number of adolescents in this situation may have limited the power of this analysis.

## Conclusion

Despite lower pre-ART CD4 and older age at initiation, adolescents attending a dedicated Saturday adolescent clinic had significantly higher retention and viral suppression rates compared to adolescents attending the standard pediatric clinic. Further studies, including randomized controlled trials, are needed to confirm our results and explore factors for intervention. Additional studies are also needed to identify factors that facilitate successful delivery of care among HIV-infected adolescents as they prepare to transition to adult care.

## Supporting information

S1 File Don McKenzie hospital’s adolescents living with HIV De-identified dataset. (CSV)
